# The effect of web-based educational interventions on mental health literacy, stigma and help-seeking intentions/attitudes in young people: systematic review and meta-analysis

**DOI:** 10.1186/s12888-023-05143-7

**Published:** 2023-09-04

**Authors:** Abouzar Nazari, Gholamreza Garmaroudi, Abbas Rahimi Foroushani, Maede Hosseinnia

**Affiliations:** 1 Department of Health Education and Promotion, Faculty of Health, Tehran University of Medical Sciences, Tehran, Iran; 2 Department of Health Education and Promotion, School of Health, Tehran University of Medical Sciences, Tehran, Iran; 3 Department of Epidemiology and Biostatistics, School of Health, Tehran University of Medical Sciences, Tehran, Iran; 4 Department of Health Education and Promotion, Faculty of Health, Isfahan University of Medical Sciences, Isfahan, Iran

**Keywords:** Mental health literacy, Stigma, Help-seeking intentions/attitudes, Young people, Intervention, Meta-analysis, Systematic review

## Abstract

**Objectives:**

This study aims to provide a systematic review and meta-analysis of the literature on The Effect of Web-Based Educational Interventions on Mental Health Literacy, Stigma and Help-seeking intentions/attitudes in young people.

**Methods:**

Articles in English published between April 1975 and February 2023 were retrieved from seven databases, leading to a total of 2023 articles identified.

**Results:**

20 studies were included after applying exclusion criteria, 10 of which were eligible for meta-analysis. Results showed that web-based educational interventions had a significant positive effect on mental health literacy knowledge (SMD = 0.70, 95% CI = [0.16, 1.25]), but not on stigma (SMD = -0.20, 95% CI = [-0.48, 0.08]) or help-seeking intentions/attitudes (SMD = 0.48, 95% CI = [-0.50, 1.46]).

**Conclusion:**

This study reviewed and analyzed the effect of web-based educational interventions on mental health literacy, stigma, and help-seeking intentions/attitudes among young people. The results showed that web-based educational interventions improved mental health literacy knowledge, but not stigma or help-seeking outcomes. The study suggested several recommendations to enhance the effectiveness of web-based educational interventions on stigma and help-seeking outcomes, such as using more rigorous designs and methods, more comprehensive and multifaceted interventions, more tailored and targeted interventions, and more collaborative and participatory approaches. The study concluded that web-based educational interventions may have a greater impact on reducing stigma and promoting help-seeking among young people, which may ultimately lead to better mental health outcomes and well-being for this population.

**Supplementary Information:**

The online version contains supplementary material available at 10.1186/s12888-023-05143-7.

## Introduction

Mental illnesses often start early in life. Research shows that 75% of them begin before age 25 and 50% before age 14 [1]. These illnesses can cause distress, disability, and negative impacts on various aspects of life, such as school, work, and relationships. They can also affect the future mental health of individuals [2, 3].

However, many adolescents with mental illnesses do not get the help they need, especially in developing countries [4, 5]. This is due to a lack of trained mental health professionals in schools, limited resources for mental health services, and low mental health literacy (MHL) among young people [5–9].

MHL is defined as “knowledge and beliefs about mental disorders that help with their recognition, management and prevention [10]. It helps people to identify mental disorders and their causes, risk factors, and treatments, and to seek help when needed [11, 12]. Unfortunately, most people, especially young people, have low MHL (e.g.) [13] and face stigma related to mental health problems [14].

However, young people’s attitudes can be changed more easily than adults [15], which makes it a good opportunity to promote MHL at this age. One of the most important factors that influence young people’s help-seeking intentions/attitudes is their recognition of mental health problems and related psychological disorders. This factor is crucial because delaying or avoiding help can have serious consequences for their mental health [12, 16, 17].

Stigma is another major barrier to help-seeking intentions/attitudes and can have worse effects than the illnesses themselves [18]. This is because stigma involves negative stereotypes and prejudice (e.g., that people with mental illness are weak, dangerous, or incompetent) that lead to discrimination and exclusion in different settings (e.g., work, health care, or social situations) [19]. People with mental illness may also internalize these stereotypes, resulting in low self-esteem, negative emotions, and behaviors that lower their quality of life [20]. In turn, self-stigma and/or fear of stigma can make individuals avoid seeking help in the form of treatment, which affects their mental health [21].

Many MHL interventions are part of other interventions and it is hard to isolate their effectiveness (for review of interventions see) [12, 22]. However, it is important to evaluate the effectiveness of Web-based MHL interventions to identify what works best and to improve the cost-benefit of any future intervention by removing ineffective components. A Web-based MHL intervention that is evidence-based and cost-effective can leverage the potential of the Internet to increase MHL at the population level and potentially improve the mental health outcomes of those living with mental illness. To our knowledge, there are only two previous systematic reviews that have examined the effects of web-based interventions on MHL, stigma, and help-seeking intentions/attitudes among young people [23, 24]. Both reviews reported mixed or limited evidence for the impact of web-based interventions on these outcomes, but they differed in their inclusion criteria, search strategy, quality assessment, and data synthesis methods. For instance, one review included only RCTs [23], while the other included both RCTs and non-RCTs [24]. One review searched only four databases [23], while the other searched seven databases [24]. One review used the Cochrane risk of bias tool [23], while the other used the Effective Public Health Practice Project quality assessment tool [24]. One review performed meta-analyses for some outcomes [23], while the other [24] performed only narrative synthesis for all outcomes [24]. Therefore, there is a need for an updated and comprehensive systematic review and meta-analysis that can provide more reliable and consistent evidence for the effectiveness of web-based interventions on MHL, stigma, and help-seeking intentions/attitudes among young people.

### Aim

We aim to conduct a systematic review and meta-analysis of interventions that target MHL, help-seeking intentions/attitudes, and stigma related to mental illness in young people aged 12 to 25 years. We treat these as separate but potentially related outcomes of intervention programs, while recognizing that MHL is a complex concept that may influence stigma and other factors. However, we distinguish between help-seeking intentions/attitudes and stigma related to mental illness to avoid the confusion and inconsistency that arises when studies measuring different aspects are grouped under the broad term of “MHL” [25].

Research question: How effective are web-based interventions in (1) improving MHL and/or (2) enhancing help-seeking intentions/attitudes and/or (3) reducing stigma related to mental illness among young people aged 10 to 25 years?

## Methods

### Search strategy

This review was conducted in accordance with the PRISMA (Preferred Reporting Items for Systematic Reviews and Meta-Analyses) guidelines [26]. To identify eligible studies 7 databases were searched: PubMed, Web of Science, Scopus, ERIC, CINAHL, Cochrane library and Embase. PubMed, Web of Science, Scopus, Embase and Cochrane library were searched directly; the others were accessed via the research platform EBSCOhost. The search was conducted February 2023 and search results were limited to English language articles published between April 1975 and 2023. We limited our search to articles published between April 1975 (the year when WHO launched its first global program on mental health) and February 2023 (the date when we conducted our search), as we assumed that web-based interventions on mental health were unlikely to exist before 1975. To avoid missing any publication, we also examined reference lists of all included studies as well as review articles. Unpublished data and grey literatures, including dissertations, congress abstracts, and patents, were not included in the current meta-analysis. In addition, we removed duplicate citations. The search term was adapted for each database. See the supplementary material 1 for an example of the search string used in PubMed. Articles identified by the database search were screened to assess relevance to the aims. In addition, Google Scholar and selected reference lists were also searched to identify additional studies of interest. This review is a registered PROSPERO review: CRD42023397689.”

### Inclusion and exclusion criteria

We included studies that met the following criteria: (1) they addressed young people aged 10 to 25 years old; (2) they were randomized controlled trials, non-randomized controlled trials, experimental or before-and-after studies; (3) they delivered a web-based intervention program; (4) they had a control group or provided an intervention as treatment as usual; (5) they assessed the help-seeking intentions/attitudes and/or the mental health-related stigma and/or MHL directly through the self-report of young people, instead of relying on information from caregivers or teachers; (6) they were published in English; and (7) they reported mean changes and their standard deviations (SDs) of outcomes for both intervention and control group or presented required information for calculating of those effect sizes.

We excluded studies that met any of the following criteria: (1) they were qualitative studies, as they were not suitable for meta-analysis; (2) they had no information about participants’ age or affiliation to the educational system (so it could not be inferred whether they were under aged); (3) they did not report results (e.g., abstracts of registered clinical trials); (4) they did not have random allocation; (5) they had an observational design; or (6) they had no control group.

### Selection of studies

Two authors (A.N and M.H) independently reviewed the relevant titles and abstracts. They obtained and scrutinized the full text of the chosen studies with another author’s (GH.G) help. They resolved any disagreements by discussion or referral to a fourth author (A.RF). Some studies were excluded as irrelevant after full-text review.

### Data extraction

The data extracted included: first author’s name, publication year, study population, sample size, participants’ sex, number of subjects in each group, age range and average age of participants, trial design, type of intervention, time of post-test, mean and SD mental health literacy, stigma and help-seeking intentions/attitudes after intervention in each group. The authors converted SEs or interquartile ranges to SDs using appropriate formulas if data were reported as such.

### Risk of bias assessment

The present study uses the QuADS tool [27] to assess the quality of nine systematic reviews of mixed- or multi-method studies on different aspects of web-based interventions for MHL, help-seeking intentions/attitudes, and stigma among young people. The QuADS tool consists of 12 criteria that cover four domains: (a) research aim/s, (b) research setting and target population, (c) study design, and (d) analytic method. Each criterion is scored from 0 to 3, and the total score is the sum of these scores. The total score ranges from 0 to 36, and indicates if the study is excellent (above 80%), good (between 50% and 80%) or low (below 50%) in quality.

### Statistical analysis

The mean change (S.D) were used to estimate the overall effect size of the intervention. If S.D was not reported in each of individual study; it was calculated using following formula: S.Dchange = square root [(S.Dbaseline2 + S.Dfinal2)-(2 × R × S.Dbaseline × S.Dfinal)]. A correlation coefficient of 0.8 was considered as R-value of the above-mentioned formula [28]. Where standard error of mean (S.E.M) was only reported, standard deviation (S.D) was calculated by S.D = S.E.M × √n (n is the number of participants in each group). Finally, in studies which only reported outcome measure in graphic form, data extraction was performed by using GetData Graph Digitizer 2.24 [29].

Estimates of effect sizes were expressed based on standardized mean difference (SMD) and 95% CI from the random-effects model. The SMD was calculated by dividing the mean difference by the pooled standard deviation of the intervention and control groups. Assessment of between-study heterogeneity was carried out using Q test and I-square (I2) test [30]. Pre-defined subgroup analysis based on time of post-test, mean age, study setting, and study quality was conducted to detect potential sources of heterogeneity. The sensitivity analysis was conducted by the one-study remove (leave-one-out) approach, to explore the impact of each study on the pooled effect size. Publication bias was evaluated using visual assessment of funnel plots and Egger’s weighted regression tests. All statistical analyses were performed using Stata software version 12 (StataCorp. College Station, Texas, USA). P < 0.05 was considered as statistically significant.

## Results

**Fig. 1:**
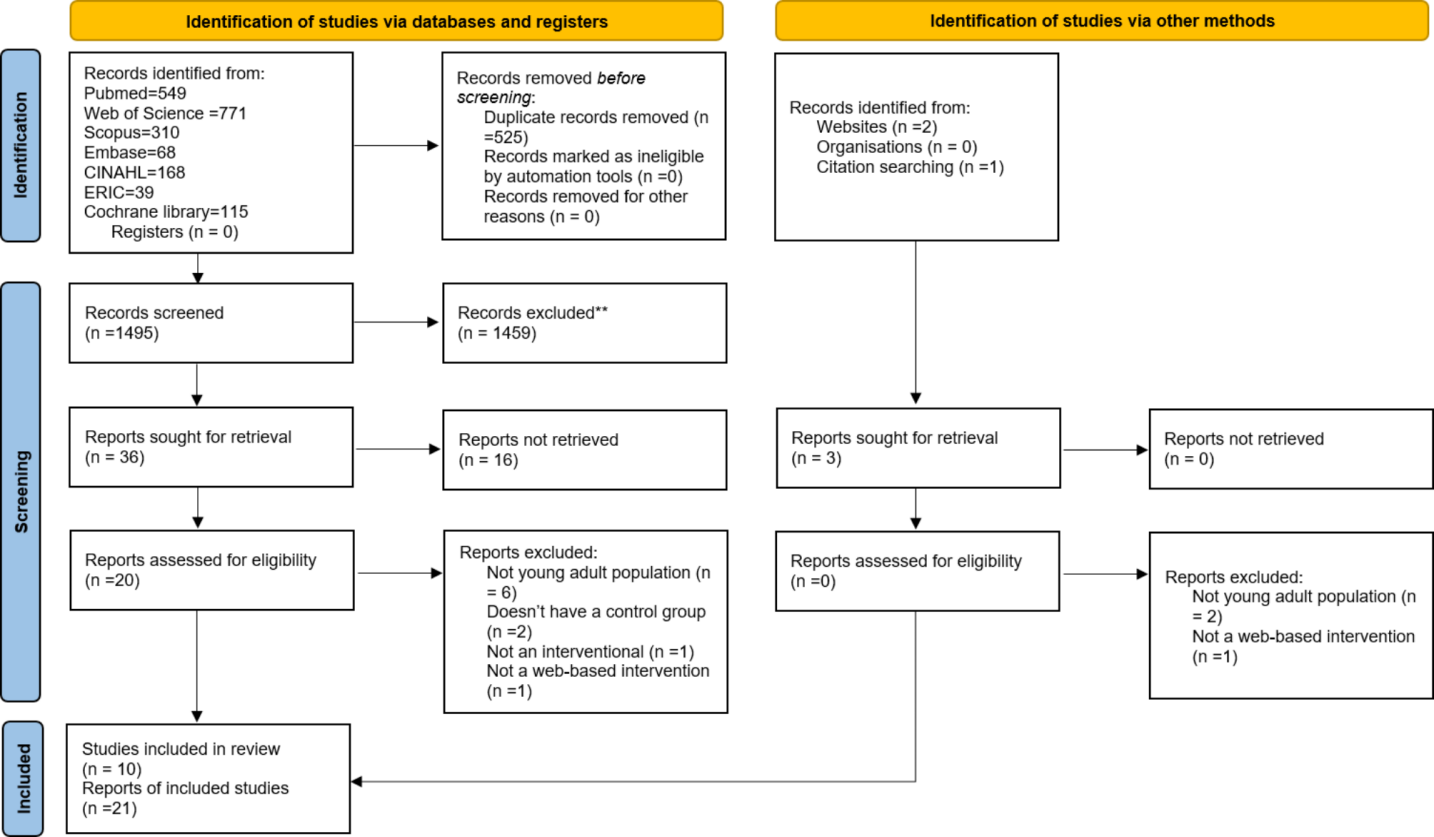
PRISMA 2020 flow diagram of papers included in the review The Effect of Web-Based Educational Interventions on Mental Health Literacy, Stigma and Help-seeking intentions/attitudes in young people, search period: 1975 to February 2023

We removed the duplicates and got 1495 records from 2020. The manual search revealed an additional 3 articles. The screening and selection process is illustrated in more detail in Fig. 1. In total, 10 studies were included for the analysis.

Two studies measured mental health literacy [31, 32], two studies measured help-seeking intentions/attitudes [33, 34], one study measured mental health literacy and help-seeking intentions/attitudes [35], one study measured mental health literacy and stigma [36], and four studies(one of the studies has two separate reports) measured help-seeking intentions/attitudes and stigma [37–39]. Of the 10 final full-text studies, 5 studies in Australia, two studies in the United States of America, one study in Ethiopia, one study in Singapore, and one study in the Netherlands. All studies included both males and females. Means and standard deviations were retrieved from studies where they were reported (n = 10).

One of the studies [37] compared two different interventions (i.e., a biological condition which comprised educational information describing the biological causes of depression vs. a psychosocial condition which comprised educational information describing the psychosocial causes of depression) and included a control group. We considered this study as two separate studies. Ten studies in total were included in the meta-analysis. Seven used randomizations [33–39], and only one study used a convenience sample (Table 1).

**Table 1:** General characteristics of the included studies

First Author (year)	Country	Study Design	setting	Gender	Participants	Length follow-up in weeks	Mean age (year)	Intervention		Outcomes
								intervention group	Control group	
Tay(2022)	Singapore	Randomized controlled trial	University	both	University Undergraduates	0, 8/5	not reported	HOPE Intervention	**-**	The Depression Literacy Questionnaire (D-Lit),The Anxiety Literacy Questionnaire (A-Lit), Personal stigma
Hassen(2022)	Ethiopia	quasi-experimental	School	both	adolescents	6	17/27	mental health curriculum	**-**	mental health literacy questionnaire(mhlq)
O’Dea(2021)	Australia	cluster randomized controlled trial	School	both	secondary school students	12	14/3	web-based mental health service (Smooth Sailing)	**-**	Mental Health Literacy Scale (MHLS), Help-seeking intentions (GHSQ)
Aller(2022)	USA	QuasiExperimental	University	both	participants across five semesters (pre-COVID-19) who were 18 years or older	25	not reported	The Mental Health Awareness and Advocacy (MHAA) Curriculum	**-**	Mental Health Awareness and Advocacy Assessment Tool(MHAA-AT)
Howard(2018)	Australia	randomized controlled trial	school	both	senior high school students aged 16 years and over	0	16	a biological condition which comprised educational information describing the biological causes of depression	**-**	General Help-seeking Questionnaire (GHSQ), Personal stigma (DSS score),Self-stigma (SSDS score)
Howard(2018)	Australia	randomized controlled trial	school	both	senior high school students aged 16 years and over	0	16	a psychosocial condition which comprised educational information describing the psychosocial causes of depression	**-**	General Help-seeking Questionnaire (GHSQ)
Tuijnman(2022)	Netherlands	Cluster Randomized Controlled Trial	school	both	adolescents	0,12, 12/8 ,25/7	13/43	game-based program Moving Stories	**-**	General Help-seeking Questionnaire (GHSQ), Dutch Depression Stigma Scale,Dutch Depression Stigma Scale,5 items from the Social Distance Scale for youths
Costin(2009)	Australia	randomized controlled trial	community	both	Young Adults	3	21/4	health e-cards	**-**	General Help-seeking Questionnaire (GHSQ)
Maybery(2022)	Australia	randomized controlled trial	community	both	Young adults aged between 18 and 25 years	6	21.83	mi.spot	**-**	General Help-seeking Questionnaire (GHSQ)
Kirschner(2020)	USA	randomized controlled trial	college	both	college students	8,12	18.99	The online interactive educational interventions lasted approximately 30 min and were created by KognitoTM, a company that develops evidence-based educational simula tions.	**-**	Attitudes toward seeking professional psychological help (ATSPPH), Perceptions of stigmatization by others for seeking help (PSOSH)

The results of the risk of bias assessment are shown in in supplementary material 2. The table shows that three studies [31, 35, 38] are excellent, four are good [33, 34, 36, 37], and two are low in quality [32, 39]. The studies that have excellent or good quality are clear about their research aim/s, research setting and target population, and study design. They get a score of 3 for these aspects. The studies that have low quality are not clear about these aspects. They get a score of 0 or 1 for these aspects. Most of the studies have problems with their analytic method. They don’t justify their choice of method, use it appropriately, or answer their research aim/s with it. They also don’t take into account the research stakeholders. These aspects need more attention in future studies.

### Mental health literacy

**Fig. 2:**
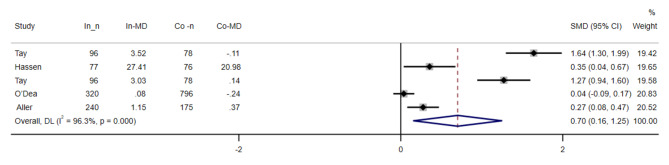
Forest plot for the effect of Web-Based Educational Interventions on mental health literacy, expressed as the mean differences between the intervention and the control groups. The area of each square is proportional to the inverse of the variance of the SMD. Horizontal lines represent 95% CIs. Diamonds represent pooled estimates from random-effects analysis

We were able to use data from five reports studies [31, 32, 35, 36], including 2,195 participants, to assess the efficacy of interventions on MHL (see Fig. 2). The results of random-effect analysis showed that interventions have a significant effect on MHL [SMD = 0.70, 95% CI = (0.16, 1.25)]. A remarkable between-study heterogeneity was observed (I2 = 96.3%, P < 0.001), By performing the analysis, it was found that other design methods (those that were not RCTs) were the source of heterogeneity(p-value > 0.10 AND I^2^ < 50%).

The results of the subgroup analysis showed a significant difference so that studies that were RCTs got lower scores than other designs. Also, in the studies that have been done in school setting, they get a lower score than other settings (supplementary material 3).

By performing sensitivity analysis, it was found that by removing each of the studies, there is no significant change in the overall result (supplementary material 4).

There was no publication bias either with Funnel’s or with Egger’s test (P = 0.057) (supplementary material 5).

### Help-seeking intentions/attitudes

**Fig. 3:**
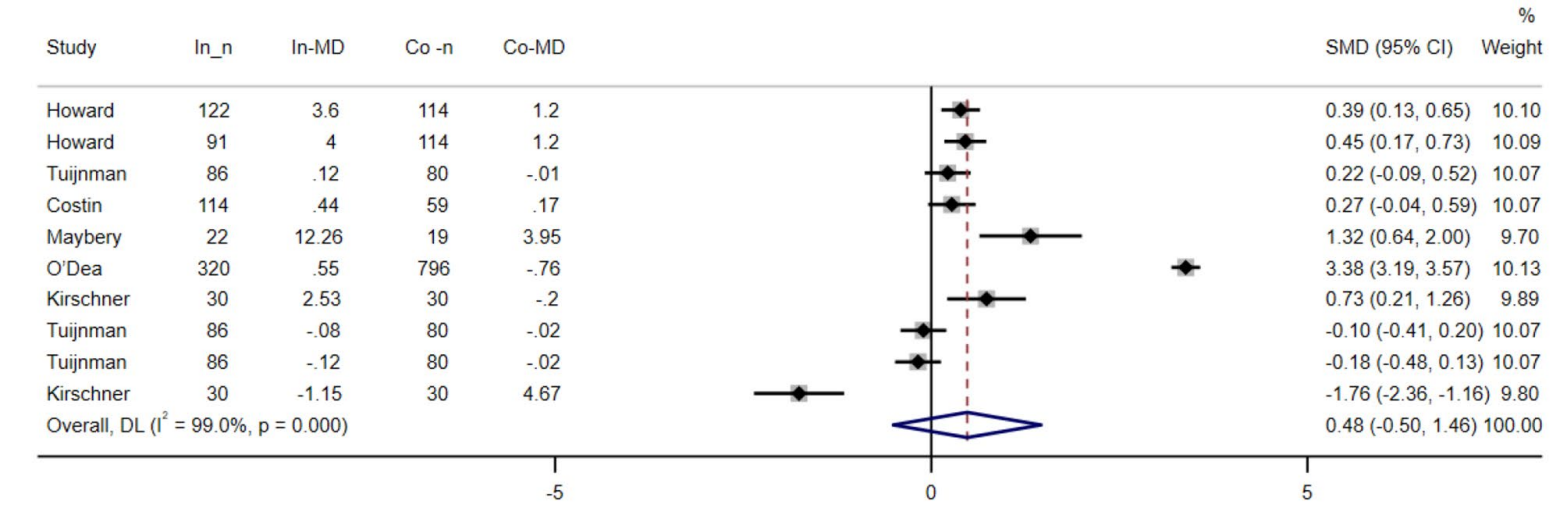
Forest plot for the effect of Web-Based Educational Interventions on help-seeking intentions/attitudes, expressed as the mean differences between the intervention and the control groups. The area of each square is proportional to the inverse of the variance of the SMD. Horizontal lines represent 95% CIs. Diamonds represent pooled estimates from random-effects analysis

We were able to use data from nine reports [33–35, 37–39], with 2389 participants, to evaluate the effectiveness of interventions on help-seeking intentions/attitudes. Help-seeking intentions/attitudes refer to the willingness or readiness to seek professional help for mental health problems. We performed a meta-analysis using a random-effects model to pool the standardized mean differences (SMDs) of help-seeking intentions/attitudes between intervention and control groups. Figure 3 shows the forest plot of the meta-analysis results. The overall pooled SMD was 0.48, with a 95% confidence interval (CI) of (− 0.50, 1.46). The p-value was 0.340. This means that the interventions did not improve help-seeking intentions/attitudes significantly.

The studies showed significant heterogeneity (I^**2**^ = 99%, P < 0.001). We performed subgroup analysis to explore the sources of heterogeneity. We used four factors: time of post-test, mean age, study setting, and study quality. These factors were chosen based on our hypothesis that the effectiveness of interventions may vary depending on how long they last, how old the participants are, where they are delivered, and how well they are conducted. There was no significant difference between subgroups based on study quality(p < 0.001), mean age (p < 0.001) and study setting (p < 0.001), but based on time of post-test (p-value > 0.10 AND I2 < 50%). There was a significant difference in that the reports collected immediately after the intervention showed higher help-seeking intentions/attitudes. The results of the subgroup analysis are shown in supplementary material 3.

We performed sensitivity analysis to examine the influence of each study on the overall pooled estimate. We omitted each study one by one and recalculated the pooled estimate and its 95% CI. The results of the sensitivity analysis are shown in supplementary material 4. None of the studies had a large influence on the overall pooled estimate, as none of them changed it substantially or made it statistically significant when omitted. This suggests that our result is robust and not driven by any single study.

We assessed publication bias using Funnel and Egger tests. The results are shown in supplementary material 5. There was no evidence of publication bias (P = 0.080), indicating that there was no systematic tendency for studies with positive or negative results to be more or less likely to be published.

### Stigma

**Fig. 4:**
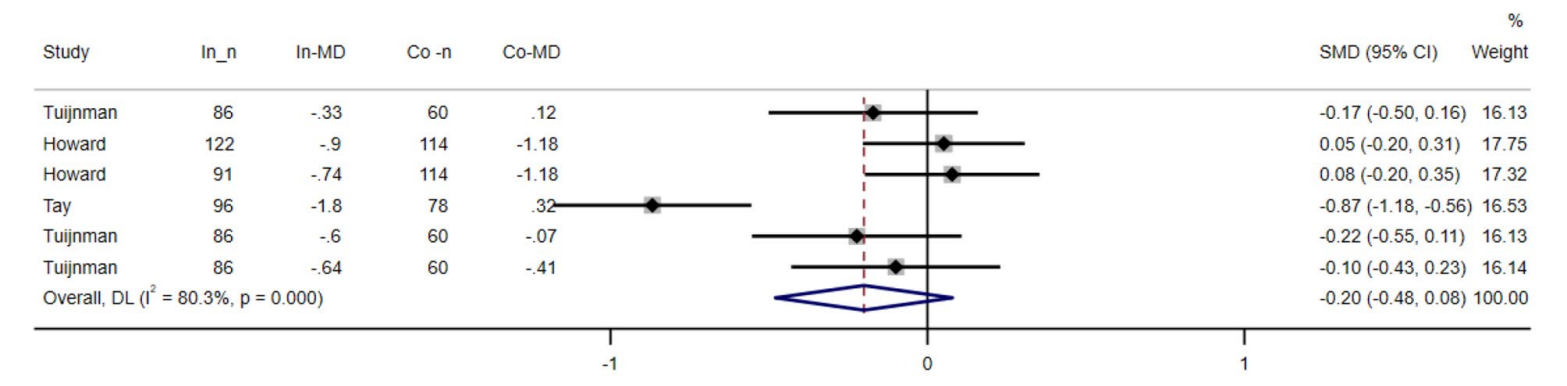
Forest plot for the effect of Web-Based Educational Interventions on Stigma related to mental illnesses, expressed as the mean differences between the intervention and the control groups. The area of each square is proportional to the inverse of the variance of the SMD. Horizontal lines represent 95% CIs. Diamonds represent pooled estimates from random-effects analysis

We were able to use data from 7 reports [36–38], including 1,053 participants, to evaluate the effectiveness of interventions in reducing stigma (see Fig. 4). The results of the random effect analysis showed that the interventions had a non-significant effect on reducing the stigma associated with mental disorders. [SMD = -0.20, 95% CI = (-0.48, 0.08)]. A significant heterogeneity was observed between the studies (I^2^ = 80.3%, P < 0.001), by performing the analysis, it was determined that the studies that measured the impact of the intervention in different countries(P = 0.891), mean ages (p = 0.874), time of post-test(p = 0.474) and study quality(p = 0.874) were a heterogeneous source. The results of the subgroup analysis showed no significant difference between subgroups based on age, time of post-test, the countries or quality assessment (supplementary material 3). By performing sensitivity analysis, it was found that removing any study did not cause a significant change in the overall result (supplementary material 4). There was no publication bias with the Funnel or Egger test (P = 0.332) (supplementary material 5).

## **Discussion**

The aim of this meta-analysis was to assess how effective web-based educational programs are at enhancing MHL, help-seeking attitudes, and stigma reduction among young people aged 10 to 25 years. We discovered that web-based programs significantly improved MHL, but had no effect on help-seeking attitudes or stigma. This indicates that web-based programs can increase young people’s awareness and understanding of mental health issues, such as the symptoms and treatments of common mental disorders and the available sources of support. However, this does not necessarily mean that young people are more willing or ready to seek professional help for their mental health problems, or that they have more positive views of people with mental disorders. This result is in line with previous reviews that have reported mixed or limited evidence for the impact of web-based programs on help-seeking attitudes or stigma among young people [40–42]. We propose four possible explanations for this finding, based on the existing literature and our own observations. First, MHL may be easier to change than help-seeking intentions/attitudes or stigma, as it involves providing factual information and correcting misunderstandings, rather than changing deep-seated beliefs and values [30]. Web-based interventions may be effective in delivering information and feedback to young people in an interactive and engaging way, but they may not be sufficient to address the emotional and social barriers that prevent young people from seeking help or reducing stigma. Second, the web-based interventions may not have been tailored enough to the specific needs and preferences of young people, who may face different challenges and opportunities to seek help or reduce stigma than adults [42]. For example, young people may have more concerns about confidentiality, accessibility, cost, or parental involvement when seeking help online or offline [44].

They may also have more exposure to negative stereotypes or discrimination from their peers, family, or school environment when dealing with mental health problems [45]. Web-based interventions may need to take into account these factors and provide more personalized and supportive content and features for young people. Third, the measures used in the studies may not have been sensitive enough to detect changes in help-seeking intentions/attitudes or stigma, as they mostly relied on self-report scales that may be biased by social desirability or lack validity [23]. For example, young people may report higher levels of help-seeking intentions/attitudes or lower levels of stigma than they actually have, or they may not understand the questions or response options well. Moreover, self-report measures may not capture the actual behaviors or experiences of young people in seeking help or reducing stigma, which may be influenced by other factors beyond their control [43].

Web-based interventions may need to use more objective and reliable measures of these outcomes, such as behavioral indicators (e.g., number of visits to online or offline services), ecological momentary assessments (e.g., real-time ratings of emotions or thoughts), or qualitative methods (e.g., interviews or focus groups). Fourth, the follow-up periods in the studies may have been too short to capture the long-term effects of the web-based interventions on help-seeking intentions/attitudes or stigma, as these outcomes may take longer to change than MHL [43]. For example, young people may need more time to process the information and feedback they received from the web-based interventions, to overcome their fears or doubts about seeking help or reducing stigma, or to encounter situations where they need to apply their skills or knowledge. Web-based interventions may need to provide more follow-up support and reminders for young people, and to evaluate their outcomes over longer periods of time.

These reasons are not mutually exclusive and may interact with each other in complex ways. Future research should address these limitations and explore the mechanisms and moderators of web-based program effects on MHL, help-seeking attitudes, and stigma among young people. The implications of our study for practice and policy are that web-based programs can be used as a cost-effective and accessible way to increase MHL among young people, but they need to be supplemented by other strategies to improve help-seeking attitudes and reduce stigma. For instance, web-based programs can be combined with school-based programs that provide face-to-face support and guidance from teachers, peers, or mental health professionals [42]. Web-based programs can also be connected with online or offline resources that offer information, advice, or referral services for young people who need further assistance [44]. Web-based programs can also be designed to target specific groups of young people who may have lower levels of MHL, help-seeking attitudes, or higher levels of stigma, such as males, ethnic minorities, or rural residents [45].

### Implication for clinical practice

Web-based interventions can increase the knowledge and understanding of mental health and mental disorders among young people who may face barriers to access and engage with traditional mental health services. Web-based interventions can also complement existing services by providing information, support, and referral options for young people who experience mental health problems or who are at risk of developing them. However, web-based interventions may not be successful in improving the attitudes and behaviors of young people toward themselves or others with mental health problems. Web-based interventions should be tailored to the specific needs and preferences of young people, and should be evaluated for their effectiveness and acceptability. Involving young people in the design and evaluation of web-based interventions can ensure that they are relevant, informed, and user-friendly. Furthermore, web-based interventions should be part of a broader and coordinated strategy to improve the mental health outcomes of young people.

### Limitation

Only articles in English were considered, which left out research published in other languages. Also, while we tried to do a comprehensive search, we might have missed some studies, including evidence from the gray literature, because we did not search for specific mental illnesses (e.g., schizophrenia) or other sources (e.g., dissertations, reports). This may introduce publication bias, as studies with positive or negative results may be more or less likely to be published in peer-reviewed journals. Also, as many of the studies were complex interventions with several parts it was hard to separate which parts influenced our target outcomes specifically. Another limiting aspect relates to outcome categorization. The studies used different tools to measure the same constructs, which makes them not easily comparable. We tried to assign each outcome to the best category to reduce heterogeneity from differences in outcome definition. Moreover, the overall risk of bias assessment showed that most studies have high or moderate risks, while few have low risk. The rather unfavorable assessment is partly due to the fact that studies do not report on all assessed aspects. We suggest that intervention studies follow reporting guidelines to overcome this information gap and offer more reliable results. Finally, given that our research question was broad and aimed to examine the effects of web-based interventions on various outcomes related to mental health literacy, stigma, and help-seeking intentions/attitudes behavior in youth, we anticipated that there would be a limited number of RCTs on this topic and excluding non-RCTs reduces the power and generalizability of our findings, so we included all RCT and non-RCT articles. However, this may introduce heterogeneity and bias in the pooled estimates, as non-RCTs are more prone to confounding, selection bias, and performance bias than RCTs, which may lead to overestimation or underestimation of the intervention effects [46]”.

## Conclusions

The evidence level of the studies included in this review varied according to their study design, quality assessment, and risk of bias. Most of the studies were non-RCTs with moderate to serious risk of bias, which limits the confidence in their findings. Only three studies were RCTs with low risk of bias [34, 36, 37], which provide more reliable evidence for the effectiveness of web-based interventions on MHL and help-seeking intentions/attitudes. These studies reported positive effects of web-based interventions on both outcomes, which is consistent with our meta-analysis results. However, our meta-analysis also included studies with high risk of bias, which may have inflated the effect sizes and introduced heterogeneity. Therefore, our findings should be interpreted with caution and more high-quality RCTs are needed to confirm the effectiveness of web-based interventions on MHL, stigma, and help-seeking intentions/attitudes in young people.

### Electronic supplementary material

Below is the link to the electronic supplementary material.

Supplementary Material 1: Search strategy

Supplementary Material 2: Risk of bias

Supplementary Material 3: Subgroup

Supplementary Material 4: Sensitivity analysis

Supplementary Material 5: Publication bias

## Data Availability

The datasets used and/or analyzed during the current study are available from the corresponding author on reasonable request.

## Abbreviations

MHL: Mental health literacy
