# Supplementary material for: The effect of web-based educational interventions on mental health literacy, stigma and help-seeking intentions/attitudes in young people: systematic review and meta-analysis
Source: BMC Psychiatry. 2023 Sep 4;23:647. doi: 10.1186/s12888-023-05143-7 (PMC10478184; doi:10.1186/s12888-023-05143-7)
Supplement: Supplementary file 1 — Supplementary Material 1: Search strategy [file 12888_2023_5143_MOESM1_ESM.docx]

**(((((((Mental[Title/Abstract]) OR (Psychological[Title/Abstract])) OR (Psychotherapeutic[Title/Abstract])) OR (Psychiatric[Title/Abstract])) AND ((((((((((Intervene*[Title/Abstract]) OR (Contact[Title/Abstract])) OR (Campaign[Title/Abstract])) OR ("health service"[Title/Abstract])) OR ("education program*"[Title/Abstract])) OR (trial[Title/Abstract])) OR (initiative[Title/Abstract])) OR (prevention[Title/Abstract])) OR (teaching[Title/Abstract])) OR (training[Title/Abstract]))) AND ((("mental health"[Title/Abstract] AND (educat* or knowledge[Title/Abstract] OR attitude*[Title/Abstract] OR literacy[Title/Abstract] OR awareness[Title/Abstract] OR curricul*[Title/Abstract] OR first aid[Title/Abstract]))) OR ("Mental health literacy"[Title/Abstract]))) AND ((((((((((((((help seek*[Title/Abstract]) OR (seek* help[Title/Abstract])) OR (seek* care[Title/Abstract])) OR (care seek*[Title/Abstract])) OR (service seek*[Title/Abstract])) OR (treatment seek*[Title/Abstract])) OR ("service use"[Title/Abstract])) OR (service utilization[Title/Abstract])) OR (Patient Acceptance of Health Care[MeSH Terms])) OR (Patient Acceptance of Health Care[Title/Abstract])) OR ("Health Care Utilization"[Title/Abstract])) OR ((Utilization[Title/Abstract] AND Health Care[Title/Abstract]))) OR ("Help Seeking Behavior"[Title/Abstract])) OR ("Help Seeking Behavior"[MeSH Terms]))) AND (((((((((Adolescen*[Title/Abstract]) OR (Adolescent[MeSH Terms])) OR (Youth[Title/Abstract])) OR (Student[Title/Abstract])) OR (Pupil[Title/Abstract])) OR (Teen*[Title/Abstract])) OR (Boy[Title/Abstract])) OR (Girl[Title/Abstract])) OR (School*[Title/Abstract]))**
