# Supplementary material for: The effect of web-based educational interventions on mental health literacy, stigma and help-seeking intentions/attitudes in young people: systematic review and meta-analysis
Source: BMC Psychiatry. 2023 Sep 4;23:647. doi: 10.1186/s12888-023-05143-7 (PMC10478184; doi:10.1186/s12888-023-05143-7)
Supplement: Supplementary file 3 — Supplementary Material 3: Subgroup [file 12888_2023_5143_MOESM3_ESM.docx]

***mental health literacy:***


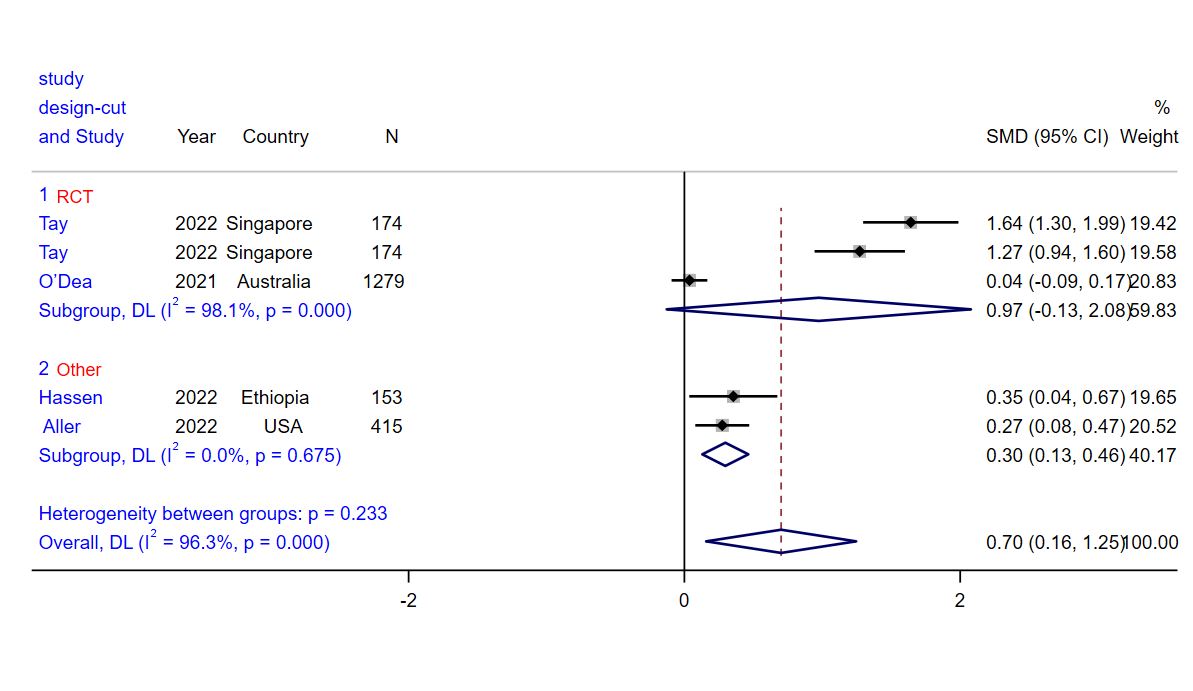


Figure 1: Subgroup analysis for the effect of Web-Based Educational Interventions on mental health literacy, expressed as the mean differences between the intervention and the control groups. The area of each square is proportional to the inverse of the variance of the SMD. Horizontal lines represent 95% CIs. Diamonds represent pooled estimates from random-effects analysis


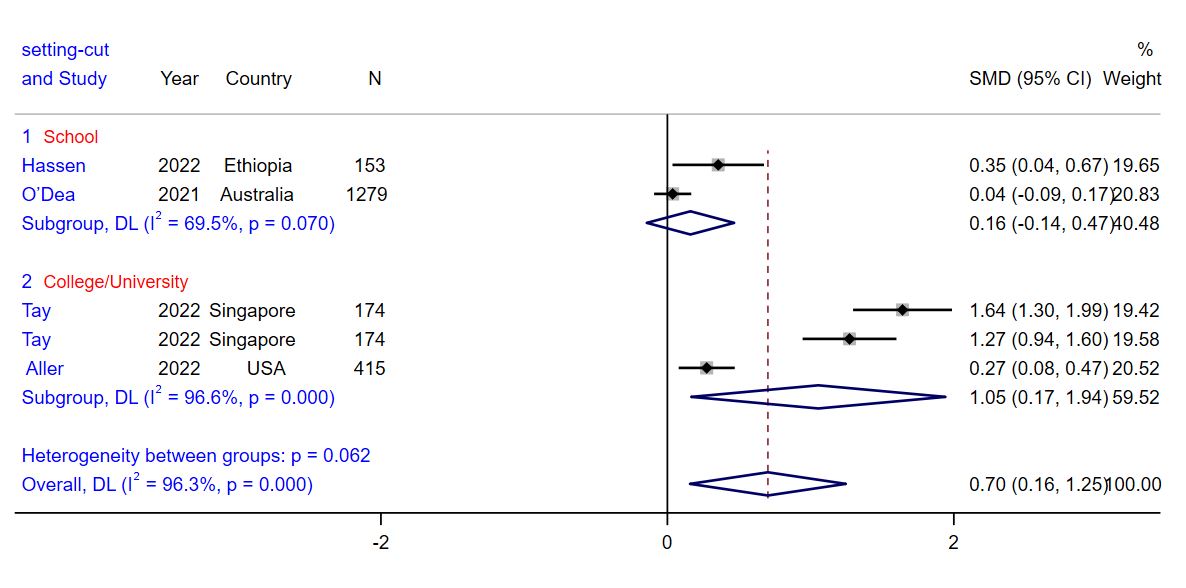
Figure 2: Subgroup analysis for the effect of Web-Based Educational Interventions on mental health literacy, expressed as the mean differences between the intervention and the control groups. The area of each square is proportional to the inverse of the variance of the SMD. Horizontal lines represent 95% CIs. Diamonds represent pooled estimates from random-effects analysis

***Help-seeking intentions/attitudes:***


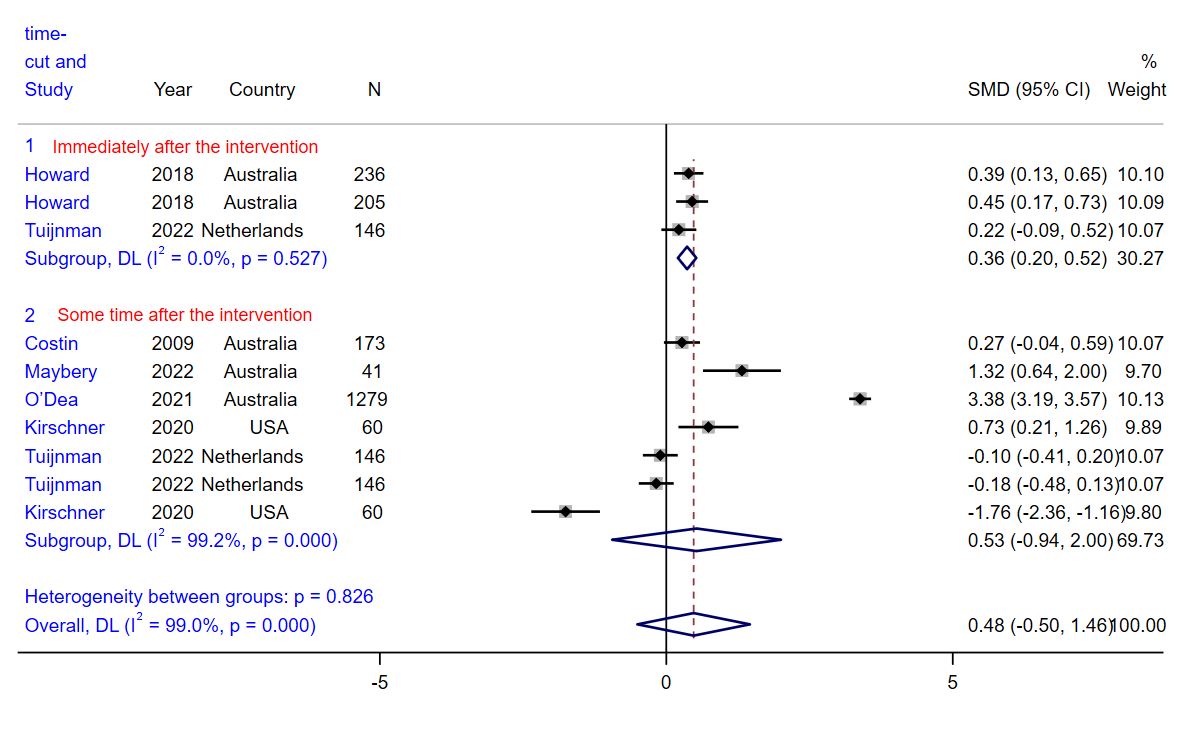


Figure 3: Subgroup analysis for the effect of Web-Based Educational Interventions on Help-seeking intentions/attitudes, expressed as the mean differences between the intervention and the control groups. The area of each square is proportional to the inverse of the variance of the SMD. Horizontal lines represent 95% CIs. Diamonds represent pooled estimates from random-effects analysis.


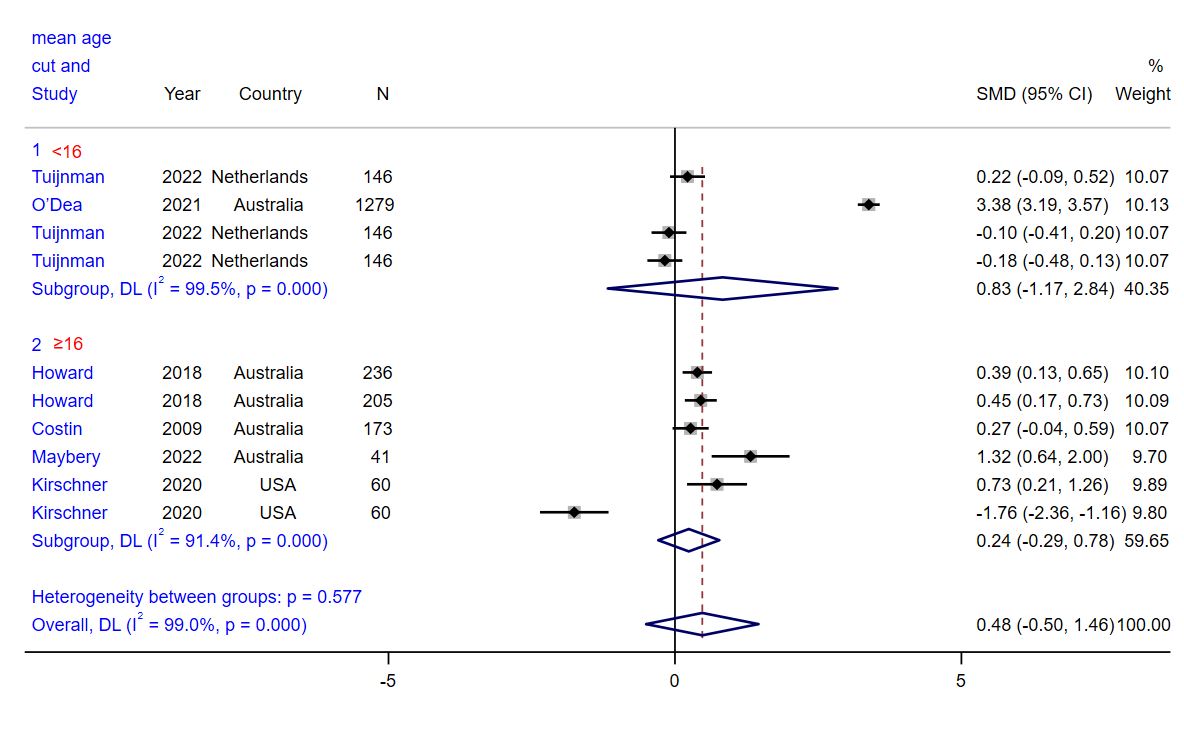
Figure 4: Subgroup analysis for the effect of Web-Based Educational Interventions on Help-seeking intentions/attitudes, expressed as the mean differences between the intervention and the control groups. The area of each square is proportional to the inverse of the variance of the SMD. Horizontal lines represent 95% CIs. Diamonds represent pooled estimates from random-effects analysis


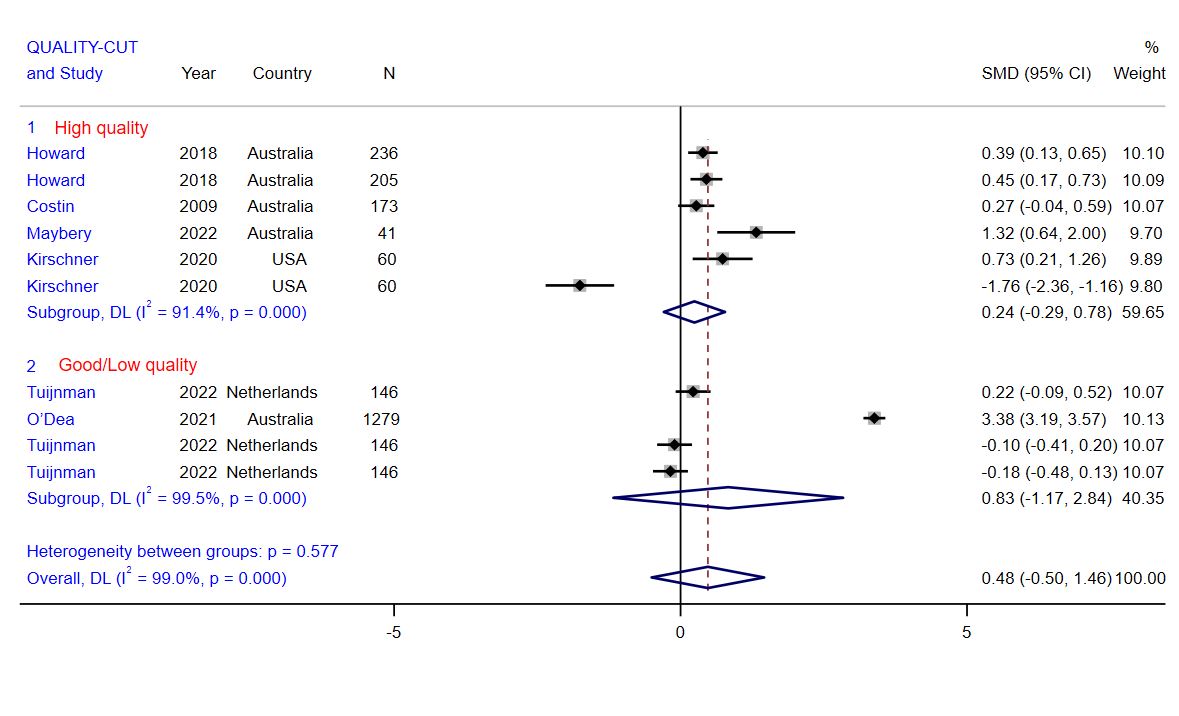
Figure 5: Subgroup analysis for the effect of Web-Based Educational Interventions on Help-seeking intentions/attitudes, expressed as the mean differences between the intervention and the control groups. The area of each square is proportional to the inverse of the variance of the SMD. Horizontal lines represent 95% CIs. Diamonds represent pooled estimates from random-effects analysis


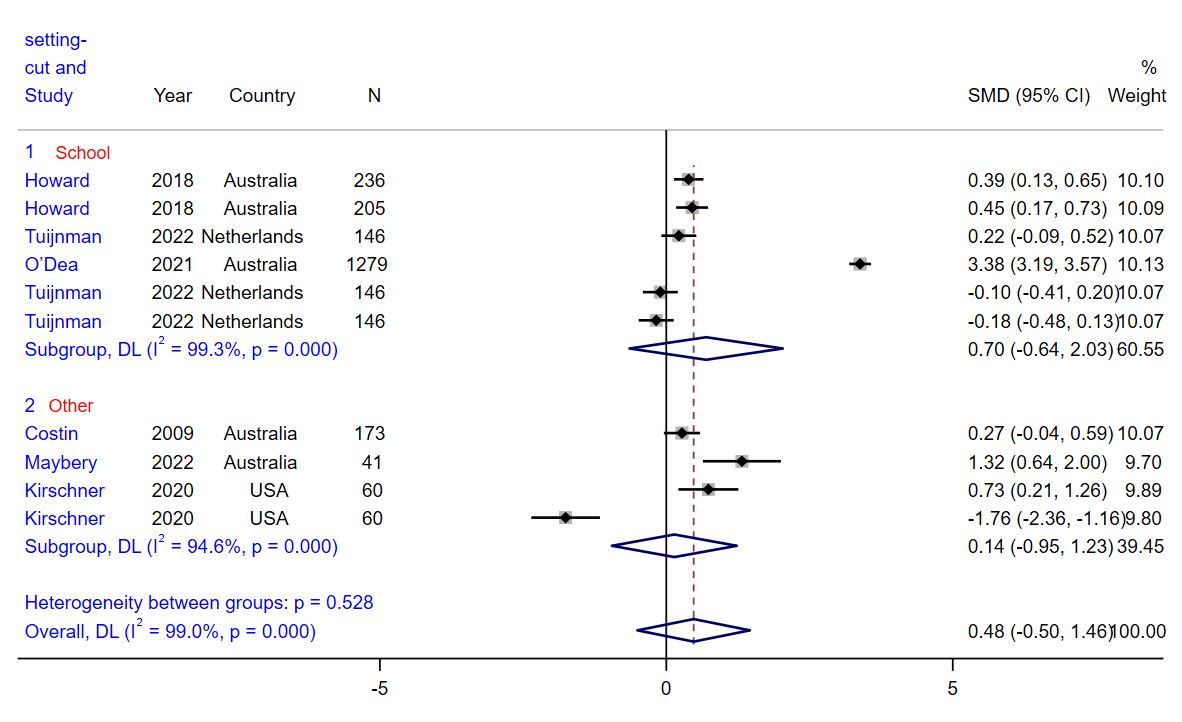
Figure 6: Subgroup analysis for the effect of Web-Based Educational Interventions on Help-seeking intentions/attitudes, expressed as the mean differences between the intervention and the control groups. The area of each square is proportional to the inverse of the variance of the SMD. Horizontal lines represent 95% CIs. Diamonds represent pooled estimates from random-effects analysis

***Stigma:***


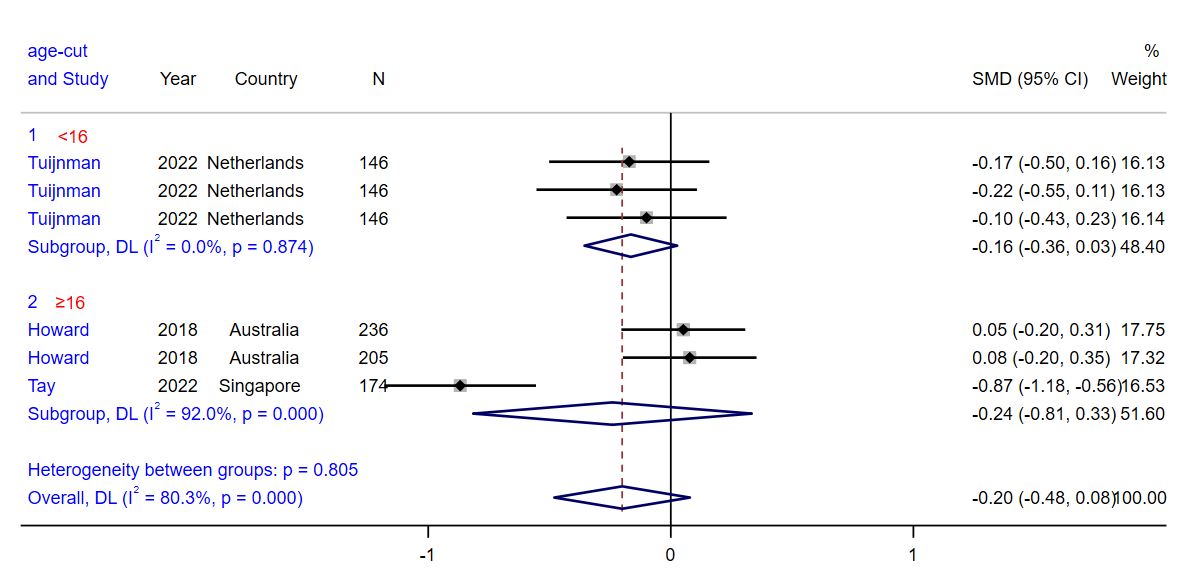
Figure 7: Subgroup analysis for the effect of Web-Based Educational Interventions on Stigma, expressed as the mean differences between the intervention and the control groups. The area of each square is proportional to the inverse of the variance of the SMD. Horizontal lines represent 95% CIs. Diamonds represent pooled estimates from random-effects analysis


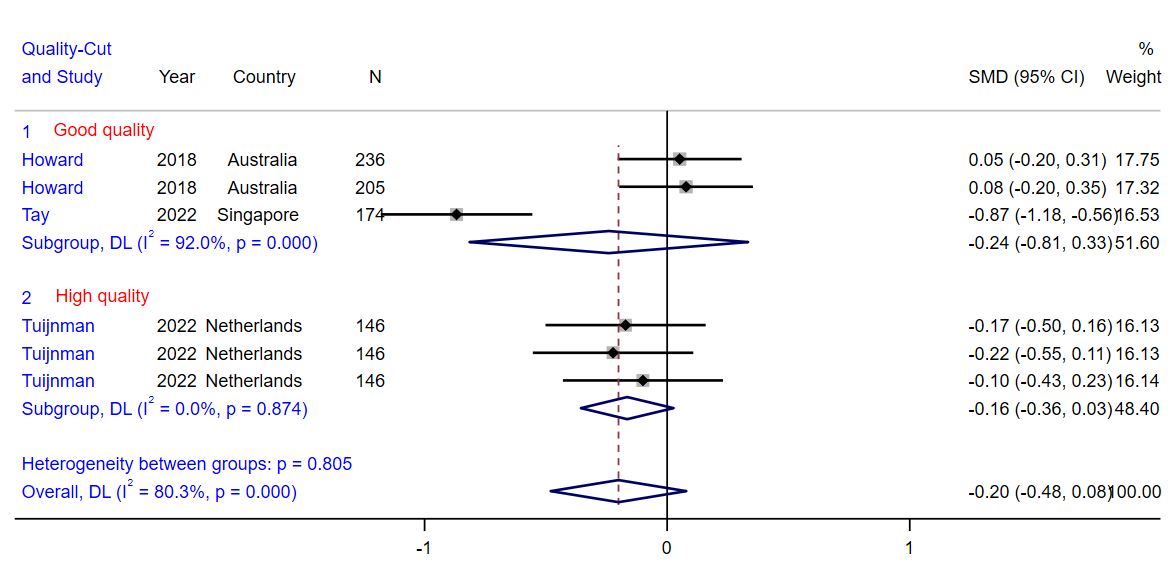
Figure 8: Subgroup analysis for the effect of Web-Based Educational Interventions on *Stigma*, expressed as the mean differences between the intervention and the control groups. The area of each square is proportional to the inverse of the variance of the SMD. Horizontal lines represent 95% CIs. Diamonds represent pooled estimates from random-effects analysis


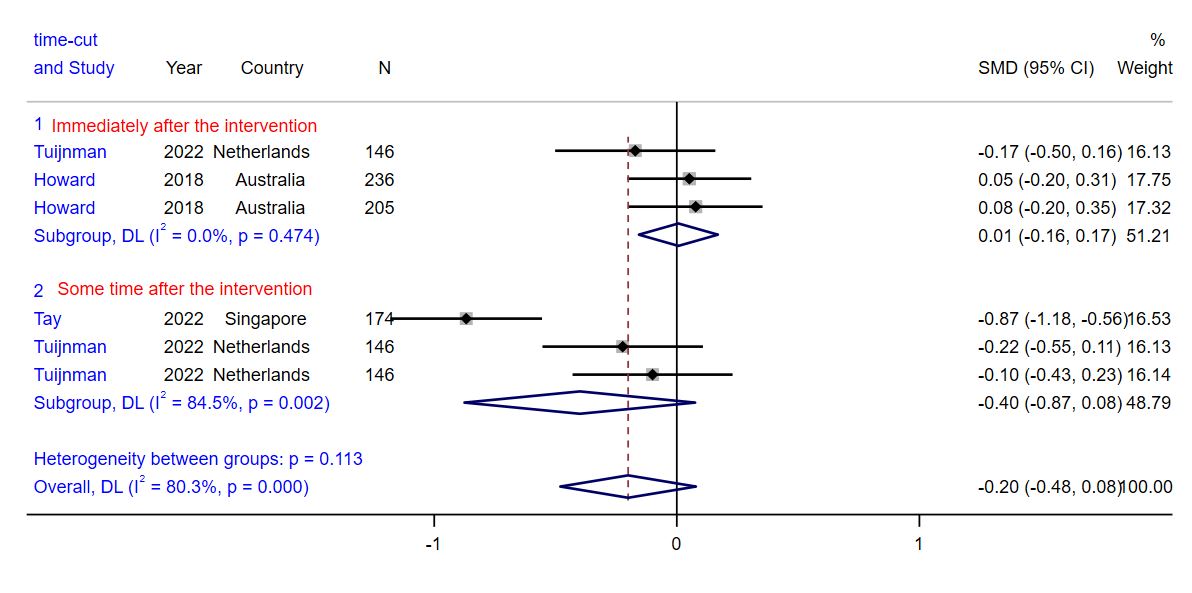
Figure 9: Subgroup analysis for the effect of Web-Based Educational Interventions on *Stigma*, expressed as the mean differences between the intervention and the control groups. The area of each square is proportional to the inverse of the variance of the SMD. Horizontal lines represent 95% CIs. Diamonds represent pooled estimates from random-effects analysis


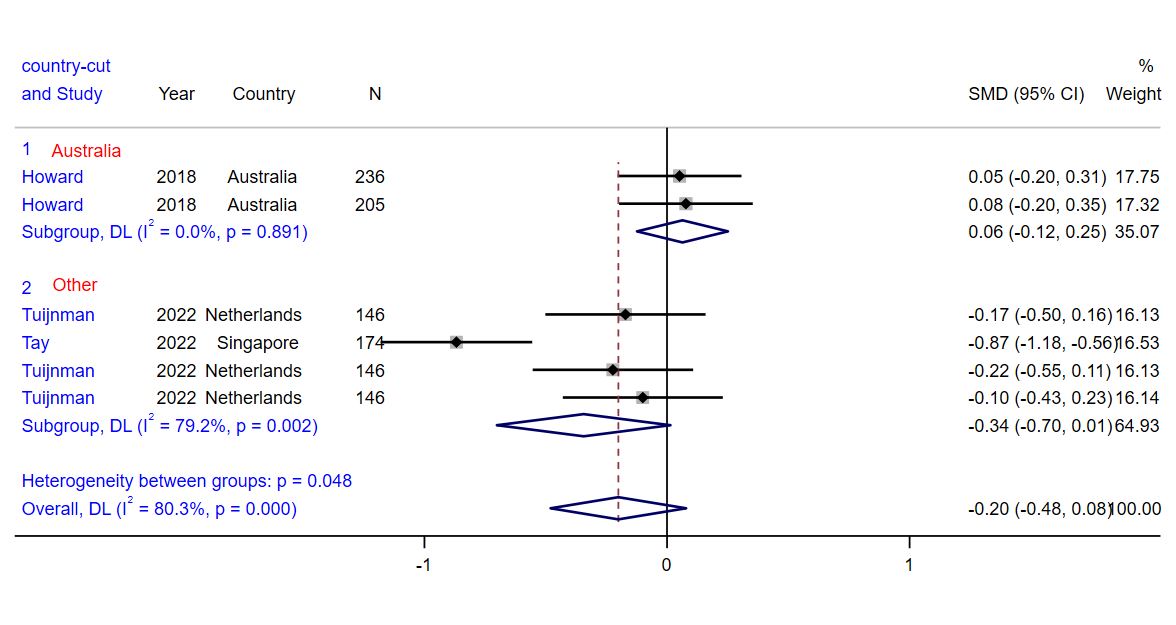
Figure 10: Subgroup analysis for the effect of Web-Based Educational Interventions on Stigma, expressed as the mean differences between the intervention and the control groups. The area of each square is proportional to the inverse of the variance of the SMD. Horizontal lines represent 95% CIs. Diamonds represent pooled estimates from random-effects analysis
