# Supplementary material for: The effect of web-based educational interventions on mental health literacy, stigma and help-seeking intentions/attitudes in young people: systematic review and meta-analysis
Source: BMC Psychiatry. 2023 Sep 4;23:647. doi: 10.1186/s12888-023-05143-7 (PMC10478184; doi:10.1186/s12888-023-05143-7)
Supplement: Supplementary file 4 — Supplementary Material 4: Sensitivity analysis [file 12888_2023_5143_MOESM4_ESM.docx]

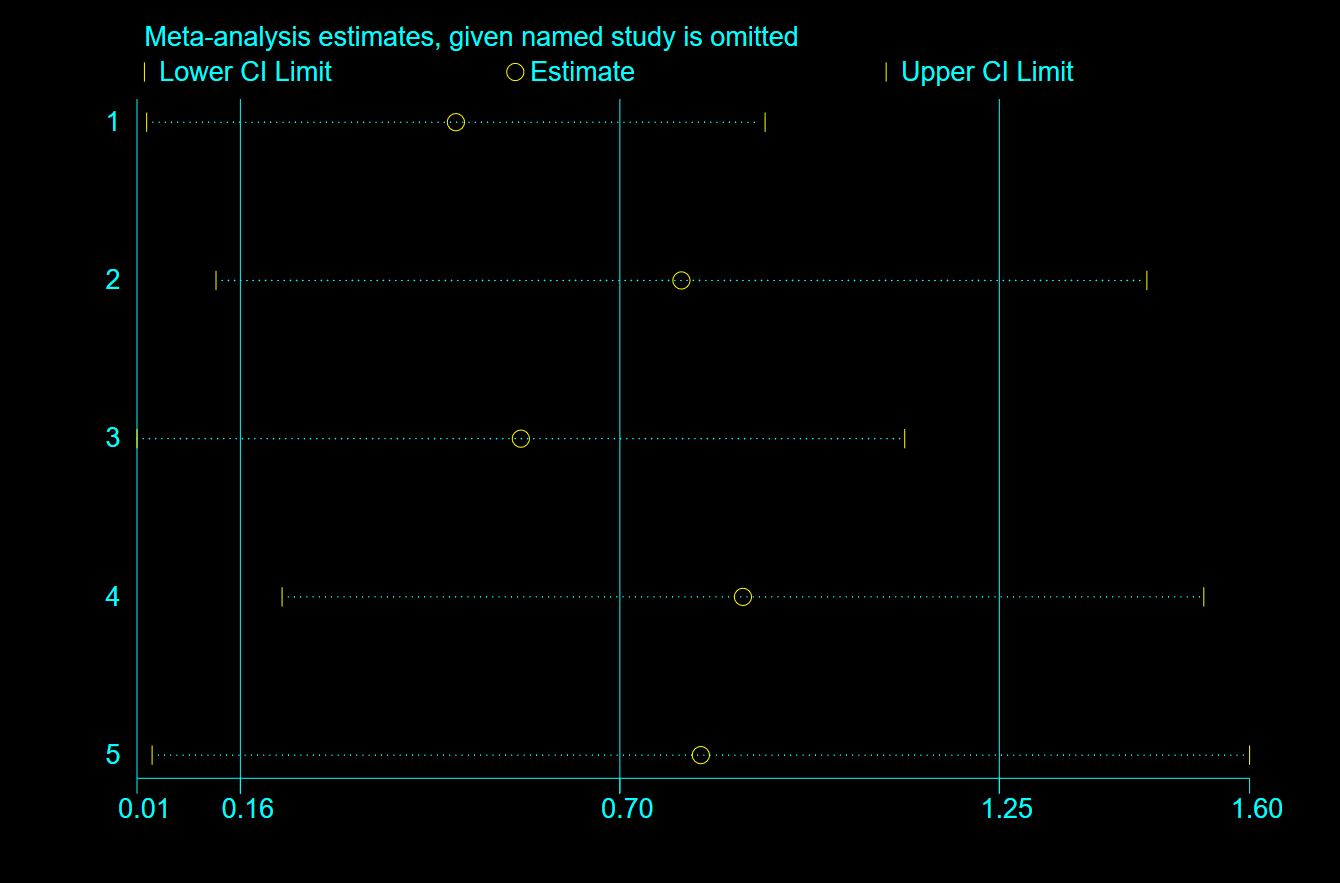


Figure 1: Sensitivity analysis of mental health literacy studies


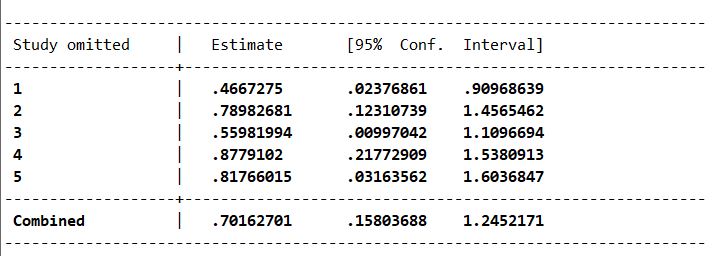


Figure 2: Sensitivity analysis of mental health literacy studies


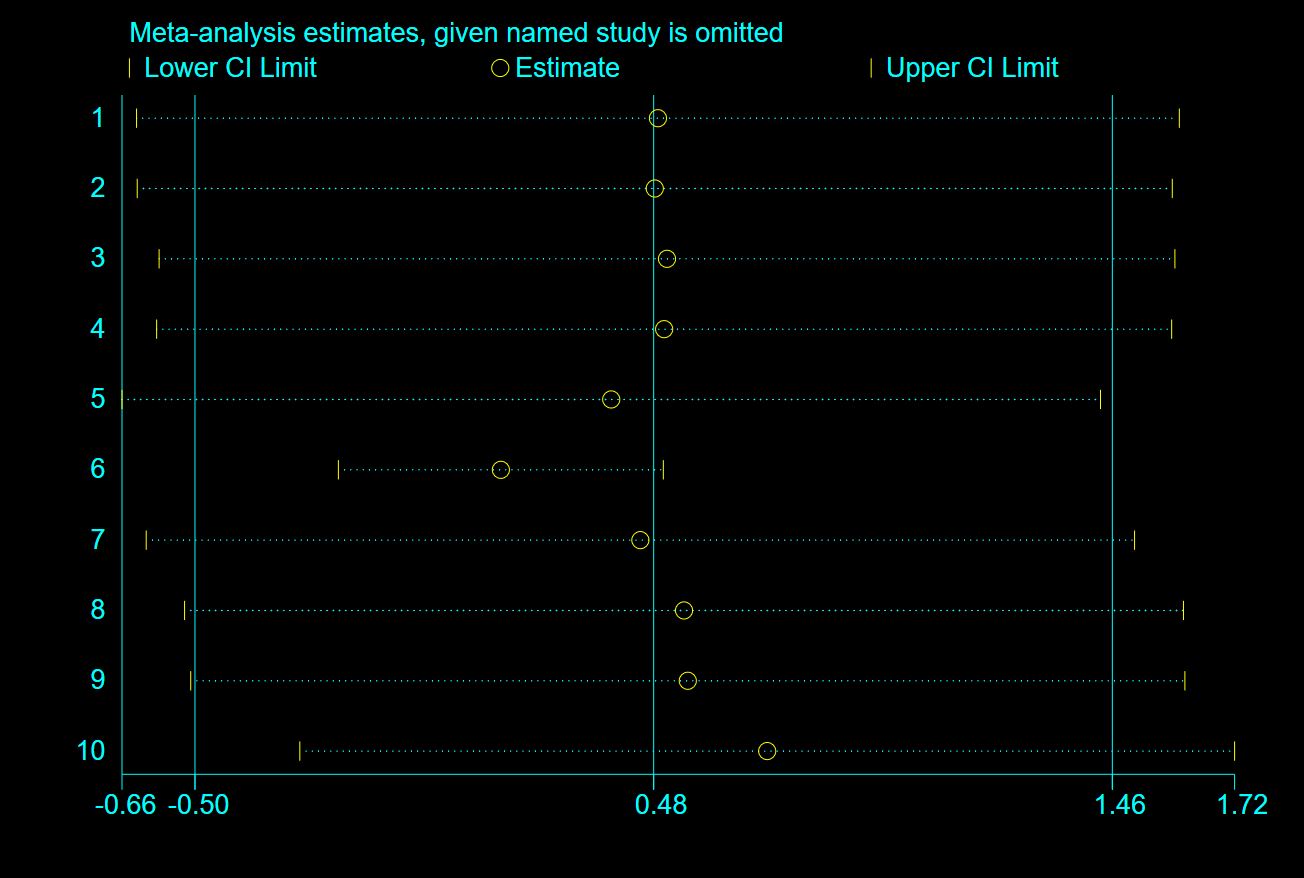


Figure 3: Sensitivity analysis of Help-seeking intentions/attitudes studies


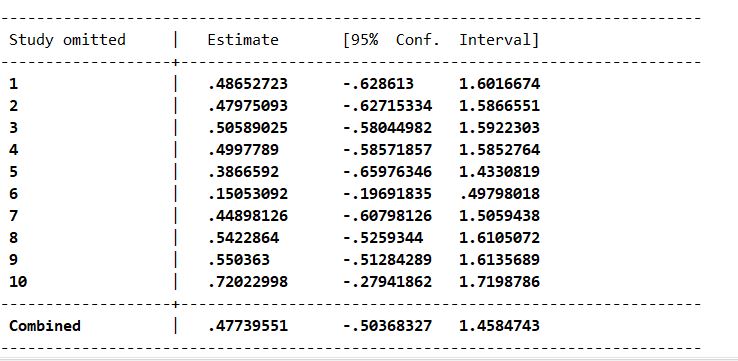


Figure 4: Sensitivity analysis of Help-seeking intentions/attitudes studies


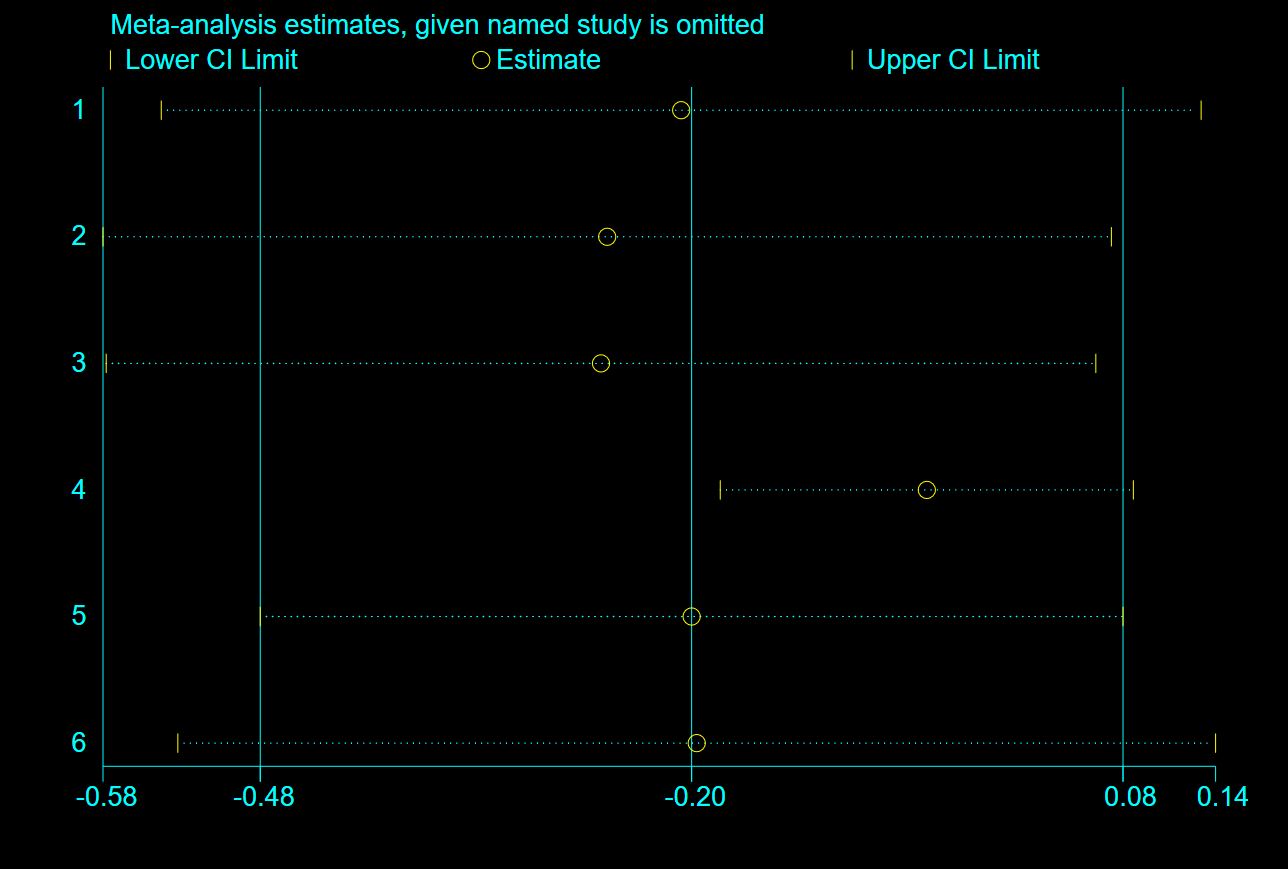


Figure 5: Sensitivity analysis of stigma studies


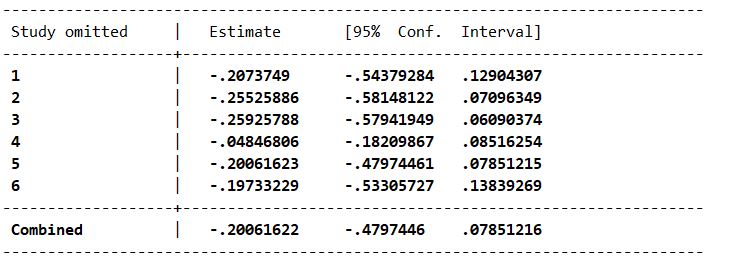
Figure 6: Sensitivity analysis of stigma studies
