# Supplementary material for: The effect of web-based educational interventions on mental health literacy, stigma and help-seeking intentions/attitudes in young people: systematic review and meta-analysis
Source: BMC Psychiatry. 2023 Sep 4;23:647. doi: 10.1186/s12888-023-05143-7 (PMC10478184; doi:10.1186/s12888-023-05143-7)
Supplement: Supplementary file 5 — Supplementary Material 5: Publication bias [file 12888_2023_5143_MOESM5_ESM.docx]

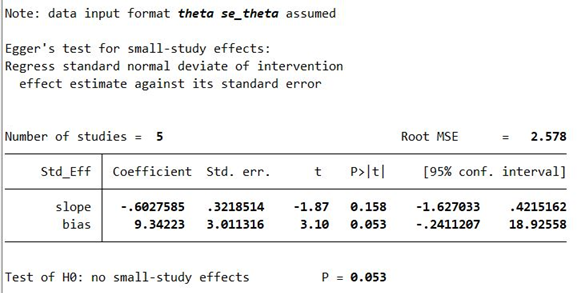


Figure 1: Publication bias results for mental health literacy studies


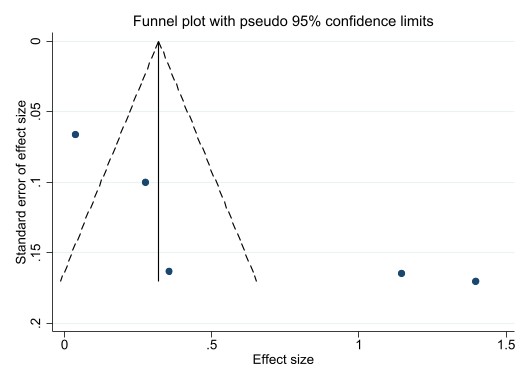


Figure 2: Publication bias results for mental health literacy studies


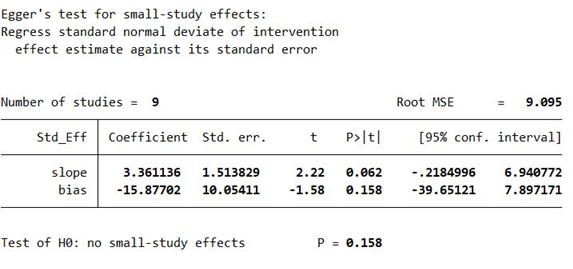


Figure 3: Publication bias results for seeking help studies


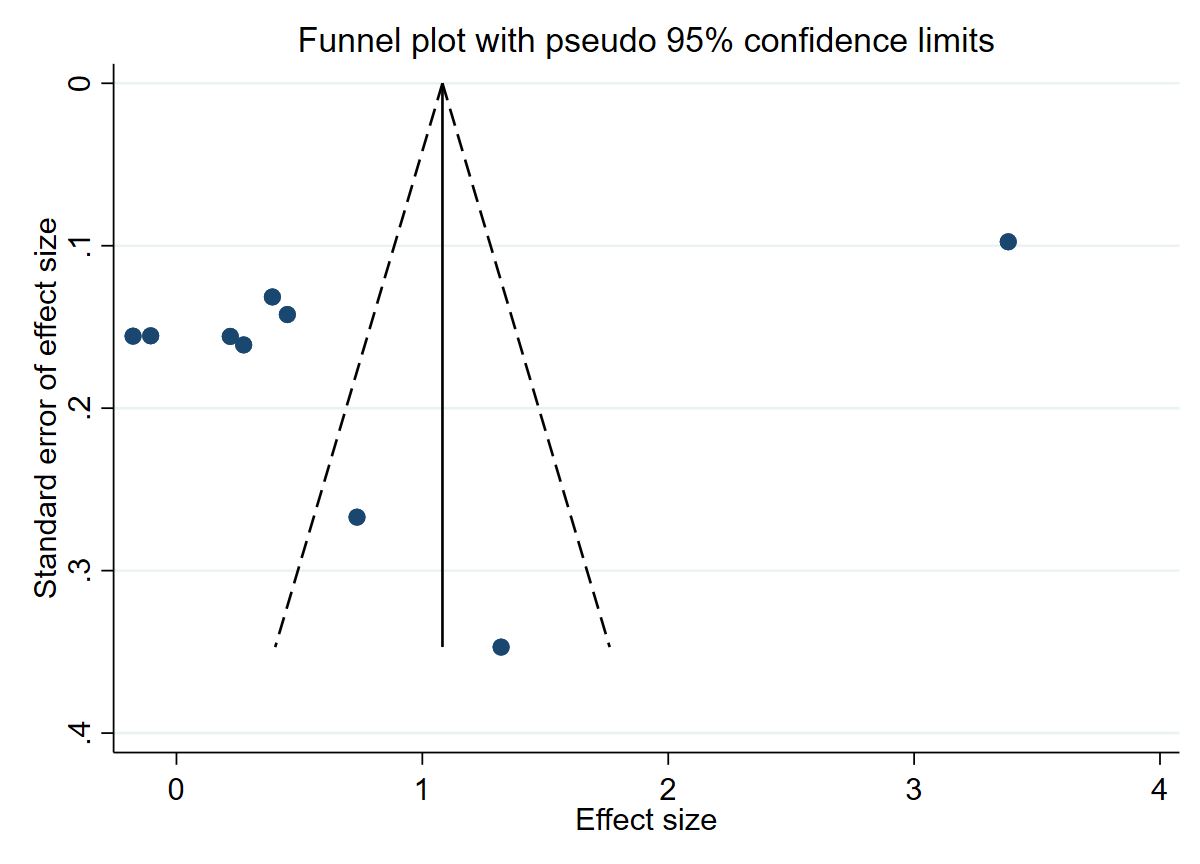


Figure 4: Publication bias results for seeking help studies


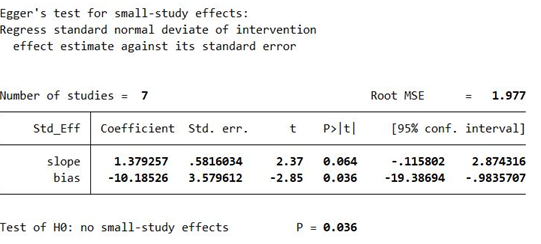


Figure 5: Publication bias results for Stigma related to mental illnesses studies


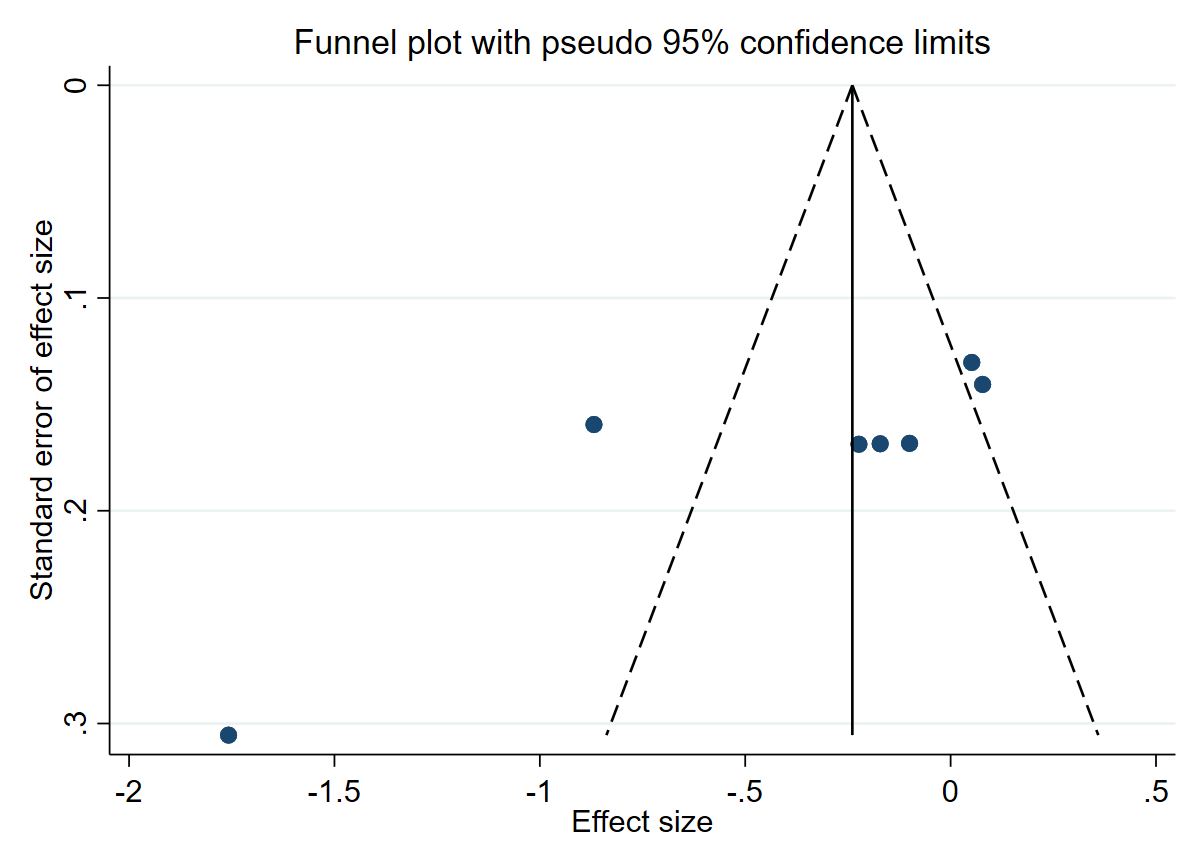


Figure 6: Publication bias results for Stigma related to mental illnesses studies
